# Antibacterial in vitro effects of preparations from Anthroposophical Medicine

**DOI:** 10.1186/s12906-016-1350-3

**Published:** 2016-09-22

**Authors:** Eva Roser, Carsten Gründemann, Inge Engels, Roman Huber

**Affiliations:** 1 Center for Complementary Medicine, Freiburg, Germany; 2 Hygiene and Molecular Laboratory, Environmental Health Sciences and Hospital Infection Control, University Medical Center Freiburg, Breisacher Str. 115B, 79106 Freiburg, Germany; 3 Center for Complementary Medicine, Department of Environmental Health Sciences, University Medical Center of Freiburg, Breisacher Str. 115B, 79106 Freiburg, Germany

**Keywords:** Staphylococcus, MRSA, Berberis radix, Berberine, Parenteral use

## Abstract

**Background:**

Medications from Anthroposophical Medicine (AM) are clinically used for the treatment of infections within a whole medical system but have not yet been evaluated regarding antibacterial effects. The aims of this study was to investigate antibacterial activity of AM medications in cell culture.

**Methods:**

Screening of AM drug registers for preparations used to treat any kind of infection and being available in dilutions ≤ D2 and without alcoholic content. Selected medications were screened for antimicrobial activity against *Bacillus subtilis*, *Escherichia coli*, *Staphylococcus aureus* and *Pseudomonas aeruginosa* using the agar diffusion method. For antimicrobial active preparations growth kinetics (drop plate method) and minimal inhibitory concentrations (MIC, macrodilution method) were determined.

**Results:**

Thirty-three preparations matched the selection criteria and were chosen for own experiments. One of them (Berberis Decoctum D2) exhibited bactericidal activities against *Bacillus subtilis* and *Staphylococcus aureus*, including methicillin resistant strains. The MIC could be determined as 5 mg/ml. The effects could be related to the content of berberine in the extract. No activity towards gram-negative bacteria was found. The other tested extracts had no antibacterial effects.

**Conclusion:**

Berberis Decoctum D2 which is used in AM to treat infections exhibits bactericidal effects on *Staphylococcus aureus*, including methicillin resistant strains.

## Background

Antimicrobial resistance is one of the major problems of modern medicine. Considering the fast development of resistance to antibiotics in bacterial species such as *Mycobacterium tuberculosis* [1] or *Staphylococcus aures* [2], it becomes obvious that there is necessity to combat further resistance expansion [3]. However there is a concerning stagnation in the development of new antimicrobial agents [4] although more and more mechanisms of bacterial resistance are being discovered. For the purpose of developing new antimicrobial agents natural substances should be considered as a promising source [5]. Besides traditional antibiotics of microbial origin like penicillin, there is clear evidence that plant-derived preparations have antimicrobial potential [6]. They are able to synergize traditional antibiotics and therefore reduce the required dosage for infection control [7, 8] and there is evidence that plant-derived preparations are able to modify mechanisms of resistance [5, 9]. Examples for commonly used phytotherapeutics in infection control are the root extracts from *Pelargonium sidoides* in the treatment of the common cold [10] or Horseradish and Tropaeolum in the treatment of urinary or respiratory infections [11–13]. Another focus of current research is the role of bacteria for the health of the human body. Referred to as the Human Microbiome it is evident today that those billions of microbes, especially located in the human gut, play a decisive role in the strengthening of health and a balanced immune system [14]. The pathogenesis of the metabolic syndrome [15] as well as allergic and autoimmune disorders such as bronchial asthma [16] or inflammatory bowel diseases [17, 18] is nowadays considered to be at least in part related to the human microbiome and especially to the gut microbiota. Antibiotics are well-known for their hurtful effects on this sensitive system of intestinal microflora. It is not uncommon that patients need microflora reconstruction after broad-spectrum antibiotic treatment. In this regard it is particularly interesting to examine substances with selective effects on the gut microflora or on bacterial strains in general: substances which have the characteristic of combatting harmful microbes but spare the physiological flora or even supporting and protecting it. Therein lies another possible role of plant-based antimicrobials in contrast to conventionally used antibiotics. Usually their potency isn’t as resounding that they would eradicate the majority of microbes of intestinal microflora and therefore provide niches for selectivity and protective potential.

Drugs from Anthroposophical Medicine (AM) have to our knowledge not yet been explored regarding their antimicrobial effects. Their manufacturing process is sophisticated and often different from manufacturing process in phytotherapy [19]. They are amongst others used to treat infectious diseases, despite they are not claimed to possess antimicrobial effects. They are instead designed to improve the self-healing capacity of the body in these diseases [20]. Nevertheless they are a possible source of new types of antimicrobial compounds. Preparations of AM are traditionally being used in German-speaking areas. The drug compendia contain approximately 1500 different medicinal products including plant-based, animal-based, mineral and combined preparations [21, 22]. The aim of this study was to investigate the antimicrobial potential of plant-based preparations of AM.

## Methods

Compendia of drugs from Anthroposophical drug manufacturers [21, 22] have been searched for preparations in dilutions ≤ D2 and free of alcoholic content. Higher dilutions have been excluded in order to guarantee that the preparations contained active substances. Alcoholic content was not accepted because it exhibits antimicrobial effects itself. Preparations with more than one plant have also been excluded in order to observe merely the effects of individual plants. Out of the remaining preparations those were chosen for proper experiments which were used for the treatment of infections in AM.

### Bacterial strains used for antimicrobial assays

*Bacillus subtilis* (ATCC 6633), *Escherichia coli* (ATCC 25922), Methicillin-susceptible *Staphylococcus aureus* (MSSA, ATCC 29213), *Pseudomonas aeruginosa* (ATCC 27853), Methicillin-resistant *Staphylococcus aureus* (MRSA, ATCC 43300) and clinical isolates of MSSA (MSSA 1883, 2114, 2289) and MRSA (MRSA 4331) from the Hygiene and Molecular Laboratory of the Institute of Environmental Health Sciences and Hospital Infection control of the University Medical Center, Freiburg, Germany were chosen for own experiments.

### Activity screening following the agar diffusion method

A bacterial suspension of one of the microbial strains listed above was produced in isotonic saline solution by visual comparison to a 0.5 McFarland turbidity standard (bioMérieux, Germany). This standard corresponds to a concentration of 10^8^ colony forming units (CFU)/ml. This solution was used to prepare bacterial smears in three layers on Columbia Blood Agar plates (Thermo Scientific, Great Britain). The test substances were then brought onto the bacterial layer in drops of 10 μl at defined positions. The agar plates were incubated for 24 hours at 37 °C.

### Growth Kinetics following the drop plate method

An overnight culture was prepared the day before by solving 1-3 colonies of a bacterial strain in 5 ml of Mueller-Hinton broth (MHB) (Merck KGaA, Germany) and by incubating this solution for 24 hours at 37 °C in a shaker (Certomat® ; B Braun, Germany) at an intensity of 100 t/min. The overnight culture was then set to a concentration of 10^8^ CFU/ml in a Turbiditymeter (Micro Scan, US) and diluted to a concentration of 10^6^ CFU/ml. Control and test samples were prepared by mixing 1000 μl of double-concentrated MHB and 1000 μl of distilled water (control) or 1000 μl of the test substance, respectively. 20 μl of bacterial suspension of 10^6^ CFU/ml were finally added to control and test samples. Up to six samples could be handled simultaneously. All samples were distributed into airtight Eppendorf tubes by pipetting 200 μl of each sample into eight Eppendorf tubes (two, each for 0, 4, 8 and 24 hours incubation). The 4, 8- and 24-hour-Eppendorf tubes were incubated at 37 °C in a shaker (Certomat®; B Braun, Germany) at an intensity of 100 t/min. The 0-hour-Eppendorf tubes were serially diluted with Mueller-Hinton broth using a microtiter plate. 5 μl of each concentration was afterwards pipetted onto Mueller-Hinton Agar-Plates (Merck KGaA, Germany). Five to six dilution steps fit the agar plate properly. In the same way was dealt with the t4-, t8- and t24-eppendorf tubes after the particular incubation times. All Mueller-Hinton-Agar-Plates were incubated at 37 °C for 24 hours. After 24 hours single colonies of bacterial growth could be counted in the areas where the former 5 μl-drops have been put on the Mueller-Hinton-Agar-Plate. The counted colonies yielded a specific growth value for each sample and incubation time.

### Determination of the Minimal Inhibitory Concentration (MIC)

The MIC was determined in CAMHB (Cation Adjusted Mueller-Hinton Broth) according to the guidelines of CLSI in macrodilution-method. For Berberis Decoctum D2 a serial dilution has been performed with a concentration of 5 mg/ml in the first and a final concentration of 9.8 μg/ml in the last tube. The initial concentration of bacteria was set to 10^6^ CFU/ml and was diluted to 10^5^ CFU/ml in the samples. The samples were incubated at 36 °C and the MIC was determined as the lowest concentration without visible bacterial growth after 24 hours of incubation.

### Statistics

All experiments on bacterial strains which play a major role in human infections and showed noteworthy effects after 24 hours have been carried out at least twice on the same bacterial strain or different clinical isolates of one bacterial species. Because the growth of bacteria in a growth kinetic assay varies to a small extend between different experiments an inhibitory effect was defined as the difference of at least 1 logarithmic unit between test sample and control. Thus only marked differences of growth were included.

## Results and Discussion

Eighty four preparations with a total of 54 different ingredients matched the inclusion criteria and have been selected out of the Anthroposophical drug registers. 33 of those were then chosen for proper experiments because they were either used for infection control in AM or because they were available in dilutions ≤ D2 (Table 1). It can be assumed that the majority of all available and eligible preparations have been screened.

**Table 1:** Preparations of AM with botanical name and dosage form which were chosen for proper experiments

Botanical name	listed preparation of AM	dosage form
Arnica montana	Arnica e planta tota (Arnica montana e planta tota ferm 33c) D2	preparation for injection
Astragalus exscapus	Astragalus exscapus D3	preparation for injection
Belladonna	Belladonna Rh D3	aqueous solution
Berberis vulgaris	Berberis, Decoctum D2	preparation for injection
	Berberis, Fructus Rh D2	preparation for injection
Betula pendula	Betula, Cortex, Decoctum D2	preparation for injection
Bryonia cretica	Bryonia D3	preparation for injection
Bryophyllum	Bryophyllum 5 %	preparation for injection
Selenicereus grandiflorus	Cactus ex herba (Selenicereus grandiflorus ex herba ferm 33d) D2	preparation for injection
Cinnamomum camphora	Camphora (Camphora aquos.) D3	preparation for injection
Cetraria islandica	Cetraria praeparata (Cetraria islandica) 2 %	preparation for injection
Chamomilla recutita	Chamomilla, Radix, Decoctum D3	preparation for injection
Cichorium intybus	Cichorium Rh D3	aqueous solution
Cydonia oblonga	Cydonia e fructibus (Cydonia oblonga e fructibus ferm 33b) D2	preparation for injection
Echinacea angustifolia	Echinacea angustifolia Rh D3	preparation for injection
	Echinacea e planta tota (E. pallida e planta tota ferm 33c) D2	preparation for injection
Gentiana lutea	Gentiana lutea Rh 5 %	aqueous solution
Geum urbanum	Geum urbanum e radice (WS: G.u. e radice ferm 33c) D3	preparation for injection
	Geum urbanum e radice D1	mother tincture
Levico	Levico (Levico water) D3	preparation for injection
Levisticum	Levisticum Rh D3	aqueous solution
Mandragora officinarum	Mandragora officinarum e radice ferm 34d, D2	preparation for injection
Nicotiana tabacum	Nicotiana e foliis (Nicotiana tabacum e foliis ferm 33b) D2	preparation for injection
Oxalis acetosella	Oxalis, Folium Rh D3	preparation for injection
Prunus spinosa	Prunus spinosa, Fructus Rh D3	preparation for injection
	Prunus spinosa, Summitates Rh D3	preparation for injection
Rosmarinus officinalis	Rosmarinus, Infusum 5 %	preparation for injection
Urginea maritima	Scilla e bulbo (Urginea maritima var. Rubra e bulbo ferm 33b) D2	preparation for injection
Solidago virgaurea	Solidago virgaurea ex herba (S. virgaurea ex herba ferm 33c) D3	preparation for injection
	Solidago virgaurea ex herba D1	mother tincture
Symphytum officinale	Symphytum e radice (S. officinale e radice ferm 34c) D2	preparation for injection
Taraxacum officinale	Taraxacum e planta tota (T. officinale e planta tota ferm 34c) D3	preparation for injection
	Taraxacum e radice (autumnale) (T.off. e radice ferm 34c) D3	preparation for injection

Out of the 33 preparations screened with the agar diffusion method, four (Berberis Decoctum D2, Betula Cortex Decoctum D2, *Solidago virgaurea* Mother Tincture and *Geum urbanum* Mother Tincture) showed antimicrobial effects. The antimicrobial activity of the mother tinctures *Solidago virgaurea* and *Geum urbanum* could be related to their pH-values (3.5 and 5.0, respectively). In a buffered assay with a neutral pH (7) the antimicrobial activity vanished. The two tinctures were therefore excluded from further experiments. It is advisable to determine the pH of plant-based preparations routinely before performing antimicrobial assays [6], not only because acidity itself has effects on bacterial growth behavior but also because it influences the impacts of active ingredients in preparations [23].

**Fig. 1:**
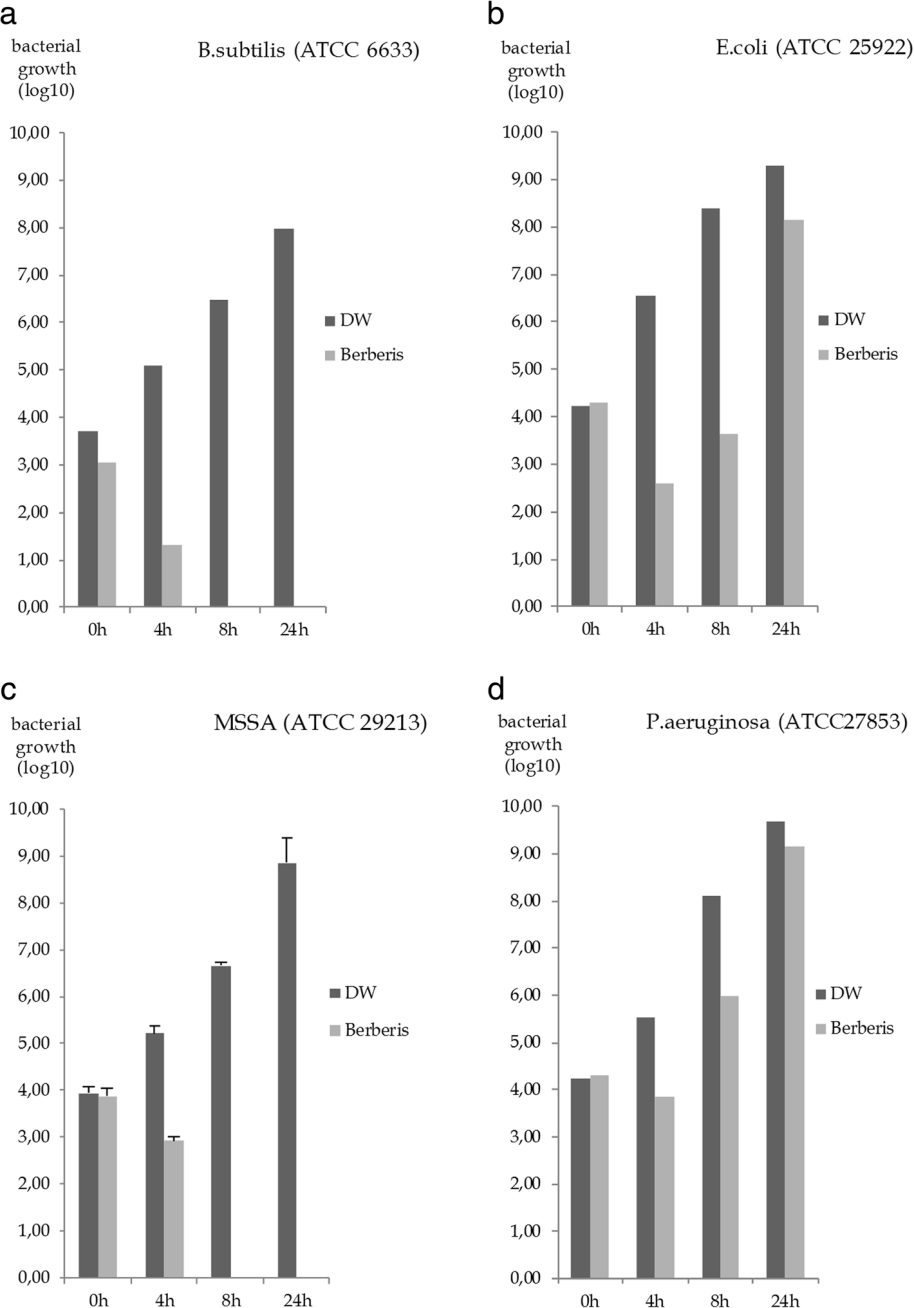
Berberis Decoctum D2 – effects on bacterial growth after 0, 4, 8 and 24 hours on logarithmic scale. Control sample: distilled water (DW); test sample: Berberis Decoctum D2. Control sample and test sample were added in a ratio of 1:2 to the culture medium. Effects are visible for *B. subtilis* (ATCC 6633) (*n* = 1) and *MSSA* (ATCC 29213) (*n* = 3) after eight and after 24 hours. Effects can be considered as bactericidal. No relevant effects after 24 hours could be found for *E. coli* (*n* = 1) and *P. aeruginosa* (*n* = 1)

All further experiments have been performed using the drop plate method. Berberis Decoctum D2 exhibited bactericidal effects on *B. subtilis* and MSSA (ATCC 29213) as shown in Fig. 1. Betula Cortex Decoctum D2 did not show any noteworthy effects and was therefore excluded from further experiments.

The bactericidal impact of Berberis Decoctum D2 could be confirmed by demonstrating the same effects on clinical isolates of MSSA (abscess-associated 1883, catheter-associated 2114, wound swab 2289) and on one clinical isolate of a multi-drug resistant strain MRSA (wound swab 4331). Because *B. subtilis* plays no major role in human infections further experiments were focused on *S. aureus* strains.

**Fig. 2:**
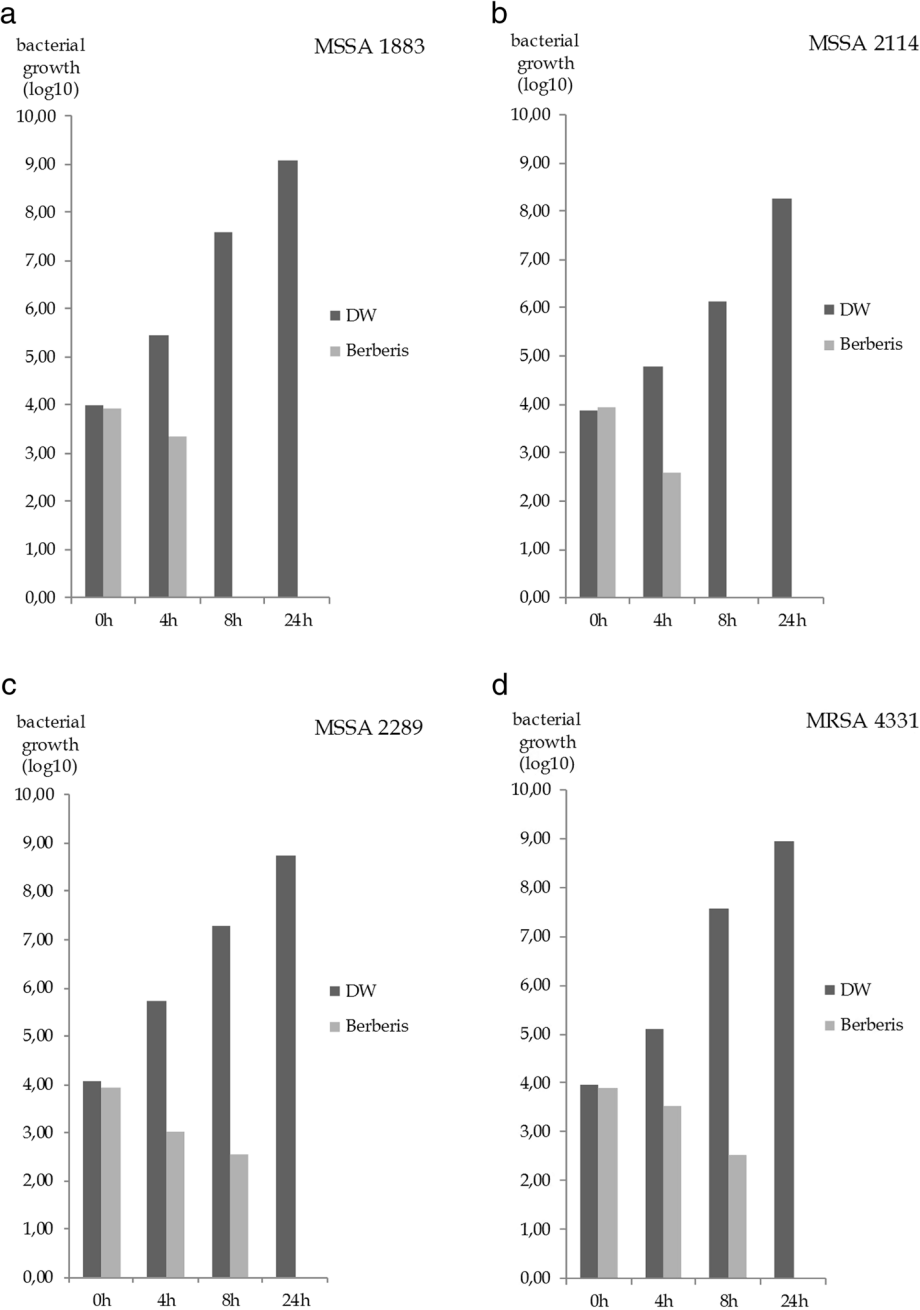
Effects on bacterial growth of *Staphylococcus aureus* strains (clinical isolates, each *n* = 1) after 0, 4, 8 and 24 hours on logarithmic scale. Control sample distilled water (DW) and test sample Berberis Decoctum D2 were added in a ratio of 1:2 to the culture medium. Bactericidal effects are visible for all *S. aureus* strains

Figure 2 shows the same outcomes for all tested *S. aureus* strains including one multi-drug resistant strain. Berberis Decoctum D2 is manufactured from the bark and roots of *Berberis vulgaris* and contains the alkaloid berberine which has been described as antimicrobial active in several publications [24]. According to the manufacturers information one ampoule of Berberis Decoctum D2 contains 10 mg of the dried drug of Berberis cortex and the bark used for manufacture of the injectable contains at least 2 % of alkaloids which can be mainly considered as berberine. During growth kinetics Berberis Decoctum D2 was used in a concentration of 1:2 to the culture medium, which equals a dosage of 5 mg/ml of the dried drug in the experiment. For MSSA (ATCC 29213) and MRSA (ATCC 43300) the MIC could be determined as 5 mg/ml. A lower dosage wasn’t able to exhibit bactericidal effects. The MIC values of pure berberine for MSSA (ATCC 29213) and MRSA (ATCC 43300) were 64 μg/ml and 256 μg/ml, respectively. These values equal approximately the proclaimed concentration of berberine in the injectable Berberis Decoctum D2 (100 μg/ml). It can, therefore, be assumed that the effects of Berberis Decoctum D2 are due to the content of berberine.

Compared to common antibiotics, the concentrations of Berberis Decoctum D2 needed for bactericidal effects are considerably higher. Interesting for systemic usability would be a concentration <100 μg/ml for plant extracts or <10 μg/ml for isolated compounds [6]. In an assay revealing the effects of Berberis Decoctum D2 on human lymphocytes we found that Berberis Decoctum D2 is inducing apoptosis in about 70 % of lymphocytes if applied in a concentration of 5 mg/ml (MIC results not shown). Systemic application in a bactericidal dosage of 5 mg/ml would therefore be toxic. In AM, however, the preparation is injected subcutaneously, close to the infected areas, e.g. around the paranasal sinuses to treat sinusitis [20]. This application might indeed induce brief antibacterial concentrations in the subcutaneous tissues. It remains an open question, whether this is relevant for the treatment with this medication and weather it improves healing properties according to the concepts of AM.

The antimicrobial effects of Berberis Decoctum D2 were selective. It worked on strains of *S. aureus* and *B. subtilis*, but spared the gram-negative strains of *E. coli* and *P. aeruginosa*. It would therefore be interesting to examine the effects of Berberis Decoctum D2 on strains of the human gut flora and emphasize on selective effects in terms of a destruction of harmful germs and simultaneous sparing of protective species. For berberine-chloride, obtained from the roots of *Coptis japonica*, such a positive selectivity on germs of the intestinal flora has already been reported [25]. Furthermore it would be interesting to examine the effects of a topical application of Berberis Decoctum D2, especially in case of colonization with multi-resistant *S. aureus* strains. For this purpose Berberis Decoctum D2 could be applied in concentrations higher than the determined MIC of 5 mg/ml. Synergistic effects of Berberis Decoctum D2 to antibiotics have not yet been investigated. Such synergistic effects have been reported repeatedly for different plant derived substances [7, 26, 27]. Regarding the fact that Berberis Decoctum D2 has, in contrast to berberine, the status of an approved drug in Germany, investigations using this plant extract would be worthwhile.

## Conclusions

Our investigations revealed that Berberis Decoctum D2 has bactericidal effects on *Staphylococcus aureus*, including methicillin resistant strains, which might be clinically useful in local application.

## Abbreviations

(CA)MHB: (Cation adjusted) Mueller-Hinton broth
AM: Anthroposophical Medicine
ATCC: American Type Culture Collection
B.: Bacillus (as in *B. subtilis*)
CFU: Colony Forming Unit
CLSI: Clinical & Laboratory Standards Institute
DW: Distilled water
E.: Escherichia (as in *E. coli*)
log: logarithmic
MHB: Mueller-Hinton broth
MIC: Minimal Inhibitory Concentration
MRSA: Methicillin-resistant *Staphylococcus aureus*
MSSA: Methicillin-susceptible *Staphylococcus aureus*
P.: Pseudomonas (as in *P. aeruginosa*)
S.: Staphylococcus (as in *S. aureus*)
